# Risk stratification of young adult survivors of cancer to estimate hospital morbidity burden: applicability of a pediatric therapy-based approach

**DOI:** 10.1007/s11764-020-00939-y

**Published:** 2020-09-16

**Authors:** Christopher Clements, Kirsten J. Cromie, Lesley Smith, Richard G. Feltbower, Nicola Hughes, Adam W. Glaser

**Affiliations:** 1 Leeds Teaching Hospitals NHS Trust, Beckett Street, Leeds, LS9 7TF UK; 2 Leeds Institute for Data Analytics, University of Leeds, Leeds, LS2 9NL UK; 3 Leeds Institute of Medical Research, University of Leeds, Leeds, LS2 9NL UK; 4 Clinical and Population Science Department, School of Medicine, University of Leeds, Leeds, UK

**Keywords:** Late-effects, Aftercare, Survivors, Deprivation

## Abstract

**Purpose:**

Children and young adults (CYA) are at risk of late morbidity following cancer treatment, with risk varying by disease type and treatment received. Risk-stratified levels of aftercare which stratify morbidity burden to inform the intensity of long-term follow-up care, are well established for survivors of cancer under the age of 18 years, utilizing the National Cancer Survivor Initiative (NCSI) approach. We investigated the applicability of risk-stratified levels of aftercare in predicting long-term morbidity in young adults (YA), aged 18–29 years.

**Methods:**

Long-term CYA survivors followed-up at a regional center in the North of England were risk-stratified by disease and treatments received into one of three levels. These data were linked with local cancer registry and administrative health data (Hospital Episode Statistics), where hospital activity was used as a marker of late morbidity burden.

**Results:**

Poisson modelling with incident rate ratios (IRR) demonstrated similar trends in hospital activity for childhood (CH) and YA cancer survivors across NCSI risk levels. NCSI levels independently predicted long-term hospitalization risk in both CH and YA survivors. Risk of hospitalization was significantly reduced for levels 1 (CH IRR 0.32 (95% CI 0.26–0.41), YA IRR 0.06 (95% CI 0.01–0.43)) and 2; CH IRR 0.46 (95% CI 0.42-0.50), YA IRR 0.49 (95% CI 0.37-0.50)), compared with level 3.

**Conclusions:**

The NCSI pediatric late-effects risk stratification system can be effectively and safely applied to cancer patients aged 18–29, independent of ethnicity or socioeconomic position.

**Implications for Cancer Survivors:**

To enhance quality of care and resource utilization, long-term aftercare of survivors of YA cancer can and should be risk stratified through adoption of approaches such as the NCSI risk-stratification model.

**Electronic supplementary material:**

The online version of this article (10.1007/s11764-020-00939-y) contains supplementary material, which is available to authorized users.

## Background

Over 80% of children and young adults (CYA) diagnosed with cancer aged 0–29 years now survive to become long-term (5 years plus) survivors [1, 2]. Up to two-thirds of long-term survivors experience at least one chronic health condition, with 40% experiencing a serious health condition, and a third living with multiple health problems as a consequence of their cancer treatment [3].

**Fig. 1:**
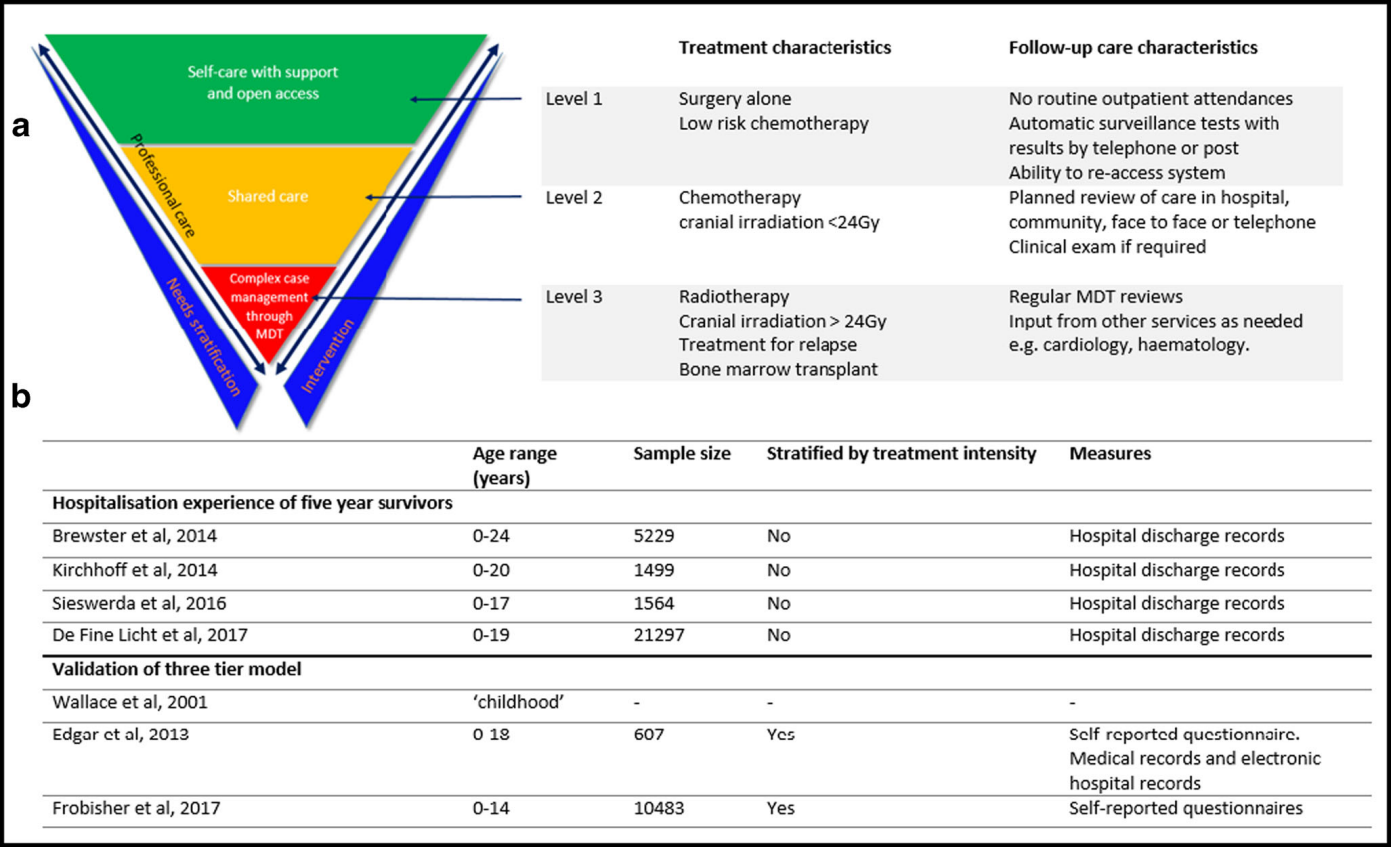
**a** NCSI three-tiered follow-up model adapted from Wallace et al. demonstrating treatment characteristics and characteristics of the follow-up model, adapted from NHS improvement strategy for children and young people survivorship [8]. **b** Summary of key papers investigating hospitalization experiences of survivors of childhood cancer and work to validate a three-tier follow-up model

The quality of life in cancer survivors varies by disease type, treatment received, and demographic factors. In order to enhance the efficiency and quality of aftercare accordingly, comprehensive risk-stratified follow-up has been implemented in the UK for all childhood (CH) survivors [4, 5]. In 2001, Wallace et al. proposed a three-tier follow-up model for survivors of childhood cancer, stratified by the intensity of cancer treatment received (Figure S1) [6]. This has been further refined by the National Cancer Survivorship Initiative (NCSI) with subsequent national roll-out and adoption of a risk-stratified three-tier follow-up model–NCSI levels of care 1, 2, and 3 [7, 8]. Level 1 represents the lowest risk, and level 3 the highest risk of morbidity associated with late effects of cancer treatment (Fig. 1a).

Subsequent population-based studies validating the NCSI three-tier follow-up model have demonstrated that treatment intensity is linked to a heightened prevalence of moderate to severe late effects [9, 10]. Other population-based work has shown 5-year survivors of cancer diagnosed before the age of 25 years are at an excess risk of acute hospital admission, compared with the general population [11–13]. Those diagnosed before their fifteenth birthday have also been observed to have an incremental increase in non-neoplastic mortality with NCSI risk level [14]. Yet to date, there have been no evaluations of the effectiveness of the three-tier model of aftercare in those diagnosed with cancer aged 18–29 years (young adults, YA) (Fig. 1b).

Individuals diagnosed with cancer aged 0–17 years (CH) are usually treated by pediatric services, with lifelong contact with specialist services mandated by the National Institute for Health and Care Excellence in the UK [5, 15]. Those diagnosed aged 18–29 (YA) are treated by adult services, where follow-up aftercare varies both structurally and in the provision of support offered [16]. However, YA cancer survivors are a distinct population with medical and psychosocial needs which may be more closely aligned to those of pediatric populations than older adults [16–18]. These specific needs may be unmet if individuals diagnosed with cancer as young adults are provided with the care provision for older adults. Most studies validating the risk stratification tool have also failed to evaluate its effectiveness across ethnic groups and levels of area-based deprivation.

This study therefore aims to investigate the applicability of the pediatric NCSI risk stratification model for predicting late-effect morbidity in a demographically diverse population of young adult cancer survivors (diagnosed 18–29 years). By conducting a thorough evaluation of the model’s effectiveness across ethnic groups and levels of area-based deprivation also, the study aims to inform comprehensive risk-stratified long-term follow-up guidelines which are applicable to all CYA cancer survivors.

## Materials and methods

### Sources of data

**Fig. 2:**
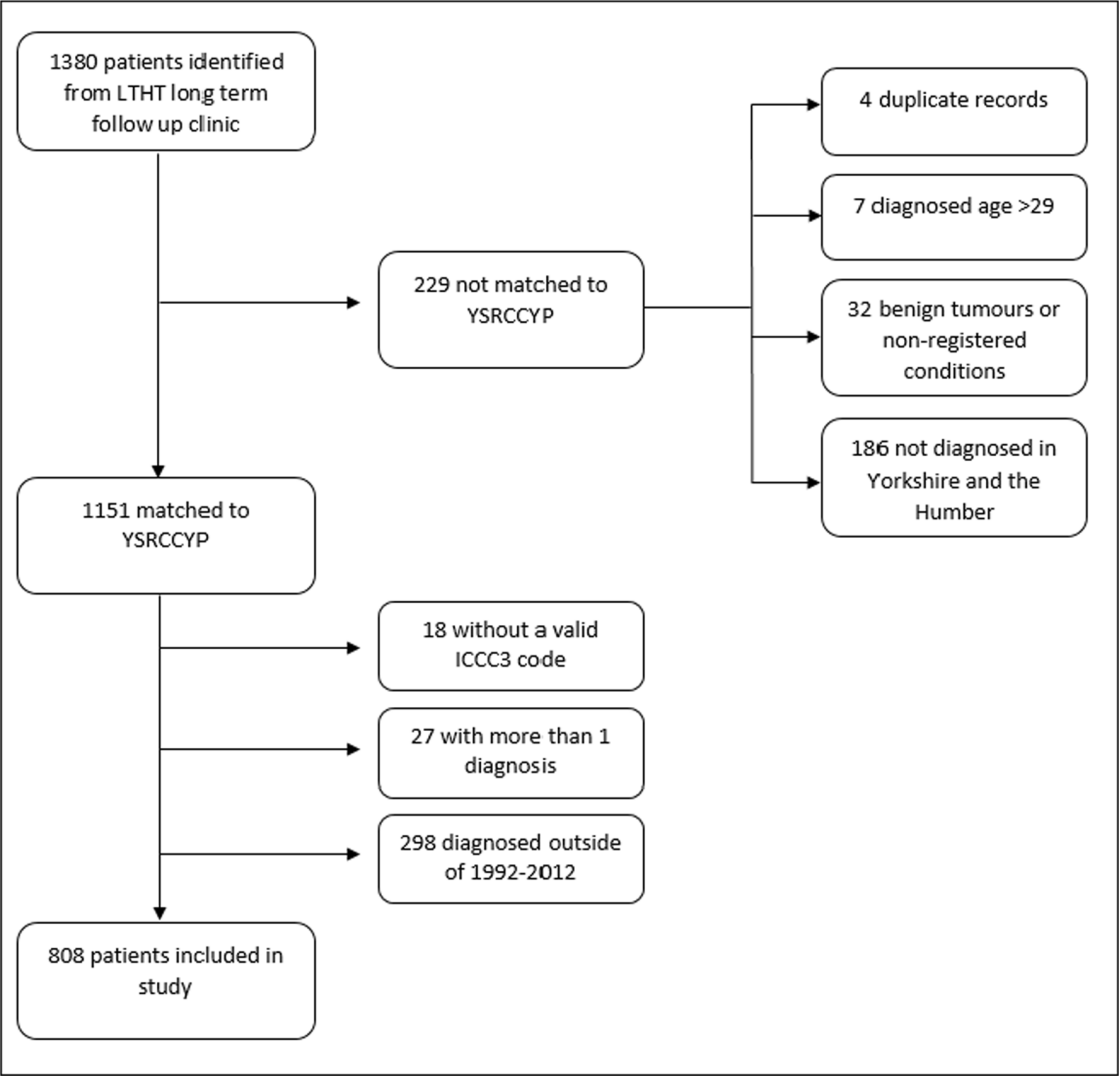
*Consort diagram for patients included in the study and the criteria not met for those excluded*. *YSRCCYP*, *Yorkshire Specialist Register of Cancer in Children and Young People*; *ICCC-3*, *International Classification of Childhood Cancer, Third Edition*

Leeds Teaching Hospitals NHS Trust (LTHT), a large regional cancer center in the North of England, maintains a database of long-term survivors whom were diagnosed with cancer and received treatment aged 0–29 years and are currently in active long-term follow-up clinics. NCSI levels have been assigned to these survivors by late-effect clinicians based on the cancer treatment received. Previous work has found a high degree of concordance between late-effect clinicians who independently assign NCSI levels [19]. This database was linked with the Yorkshire Specialist Register of Cancer in Children and Young People (YSRCCYP), a unique population-based dataset capturing clinical and epidemiologic information on 0–29 year old’s diagnosed with cancer whilst resident in the Yorkshire and Humber region, comprising clinical, patient, and sociodemographic variables [20]; record-level matching was carried out using National Health Service (NHS) number and name. The survivor cohort was defined using data drawn from the YSRCCYP, restricting the LTHT follow-up database to all 5-year survivors diagnosed aged 0–29 years between 1992 and 2012 (Fig. 2).

Hospital activity was utilized as a proxy for morbidity due to late effects of cancer treatment [21]. Hospital admissions data were linked to the YSRCCYP via an extract from Hospital Episode Statistics (HES), a data warehouse containing details of all admissions at NHS hospitals in England maintained by NHS Digital. HES records were available from April 1997 to March 2017 [22]. Linkage based on NHS number, date of birth, gender, and postcode was achieved and conducted by NHS Digital using their standard deterministic algorithm.

Information extracted from HES included the total number of hospital admissions and length of stay for each admission with the aim of investigating three main outcomes: time to first hospital admission, total number of all-cause hospital admissions, and cumulative length of hospital stay per person. Inpatient HES are recorded as a series of *Finished Consultant Episodes* which represent a period of care under a particular consultant specialty at a single hospital provider. A patient’s whole stay in hospital is known as a spell, and a spell may contain more than one episode if a patient is treated under more than one consultant during their admission. Number of admissions was based on the number of continuous inpatient spells (CIP). CIP is a continuous period of care within the NHS, regardless of any transfers between hospital providers and the spell ends when the patient dies or is discharged from hospital [23].

Ethnicity information was extracted from HES which records ethnic groups based on census categories (White, South Asian, or Other). These categories reflect the ethnic distribution of the West Yorkshire population where LTHT is situated [24]. Where ethnicity information was conflicting or incomplete, *Onomap* naming algorithms were used, in line with previous approaches [25]. The “Other” ethnic group was excluded from analysis due to low numbers, as were 10 missing values for ethnicity.

Townsend area level deprivation scores were obtained by applying 2001 National Census data [26] based on residential postcode at the time of diagnosis to the survivor cohort. Townsend deprivation scores were assigned based on the level of unemployment, non-car ownership, non-home ownership, and overcrowding [27]_._ Townsend scores were then divided into fifths. Due to the relatively low numbers within deprivation fifths 1 and 2, they were combined into a single group representing the least deprived survivors for analysis. The cohort was stratified by deprivation fifths as a whole and by NCSI levels to investigate the impact of deprivation on hospital activity.

The null hypothesis assumed that there will be no difference in the hospital activity patterns of 5-year cancer survivors across and within NCSI levels of care.

### Statistical methods

Analyses were performed both on the survivor cohort as a whole and stratified by age at diagnosis (CH, 0–17 years, and YA, 18–9 years) to investigate differences in hospital activity between the age groups by NCSI level. This method of analyses allowed us to verify the validity of the 3-tier NCSI model in our pediatric survivors diagnosed 0–17 years, the population for which it was developed, before testing it’s applicability for the predicting hospital activity of those diagnosed 18–29 years.

The follow-up period began 5 years from the date of diagnosis until date of death, emigration or 31st March 2017, whichever occurred first. The time to first admission was defined as the time between original diagnosis and their first hospital admission. Time to first admission was calculated starting from 5 years after the diagnosis date. Data regarding time to first hospital admission and total length of stay per person were positively skewed and consequently, median and interquartile range (IQR) are presented.

Poisson regression was used to model the impact of NCSI level on the total number of admissions and cumulative total length of hospital stay per person, with incidence rate ratios (IRR) used to derive effect estimates, adjusted for age at diagnosis (as a categorical variable: those diagnosed < 18 and > 18 years) and sex. A series of age-and sex-adjusted Poisson regressions were used to model the impact of deprivation on the total number of hospital admissions and cumulative length of stay, for all ages combined (age of diagnosis included as a linear effect). Separate models were also run stratified by each NCSI level. Crude admission rates were calculated for those diagnosed in childhood and as young adults by dividing total admissions per person by total follow-up period per person. Poisson regression was used to model the relationship between NCSI levels and hospital admissions and cumulative total length of hospital admissions when stratified by age at diagnosis (CH versus YA) and adjusting for sex and ethnicity. Kruskal-Wallis tests were used to determine if there were any statistically significant differences between NCSI levels on the outcome variable of interest.

Tests of interaction were explored between deprivation, sex, and age using the Bayesian Information Criterion (BIC) [28]_._

## Results

A total of 808 5-year survivors diagnosed with cancer aged 0–29 years were included in the study (Fig. 2) (CH: *n* = 668 survivors, YA: *n* = 140). The demographic and diagnostic information of study participants are presented in Table 1. The number of survivors allocated to NCSI level 1, 2, and 3 was 51 (6.3%), 447 (55.3%), and 310 (38.4%) respectively. The median person years of follow-up was similar across NCSI levels (level 1 = 11.2 person years, (IQR 5.3-15.3); level 2 = 10.4 person years (IQR 6.5–14.7); level 3 = 11.6 person years (IQR 7.0–15.9)).

**Table 1:** Demographic characteristics of all 5-year cancer survivors included in the study by National Cancer Survivorship Initiative (NCSI) risk stratification levels

			NCSI Risk Stratification Level					
	All participants		Level 1		Level 2		Level 3	
	*n*	(%)	*n*	(%)	*n*	%	*n*	%
	808	-	51	6.3	447	55.3	310	38.4
Sex								
Male	496	61.4	34	66.7	263	58.9	199	64.2
Female	312	38.6	17	33.3	184	41.2	111	35.8
Ethnicity								
White	710	89.0	45	90.0	382	86.4	283	92.5
South Asian	74	9.3	4	8.0	49	11.1	21	6.9
Other	14	1.8	1	2.0	11	2.5	2	0.7
Townsend deprivation fifth								
1 (least deprived)	37	4.6	2	3.9	23	5.2	12	3.9
2	103	12.8	9	17.7	46	10.2	48	15.5
3	191	23.6	6	11.8	116	26.0	69	22.3
4	169	20.9	10	19.6	90	20.1	69	22.3
5 (most deprived)	308	38.1	24	47.1	172	38.5	112	36.1
Year of diagnosis								
1992–1996	199	24.6	13	25.5	100	22.4	86	27.7
1997–2001	240	29.7	13	25.5	128	28.6	99	31.9
2002–2006	225	27.9	14	27.5	140	31.3	71	21.9
2007–2012	144	17.8	11	21.6	79	17.7	54	17.4
Age at cancer diagnosis (years)								
0–4	188	23.3	15	29.4	101	22.6	72	23.2
5–9	171	21.2	7	13.7	102	22.8	62	20.0
10–14	210	26.0	13	25.5	116	26.0	81	26.1
15–19	142	17.6	7	13.7	82	18.3	53	17.1
20–24	59	7.3	6	11.8	29	6.5	24	7.4
25–29	38	4.7	3	5.9	17	3.8	18	5.8
Time from diagnosis (years)								
5–9	65	8.0	3	5.9	36	8.1	26	8.4
10–14	184	22.8	16	31.4	109	24.4	59	19.0
15–19	248	30.7	10	19.6	151	33.8	87	28.1
20–24	222	27.5	17	33.3	103	23.0	102	32.9
> 25	89	11.0	5	9.8	48	10.7	36	11.6
ICCC-3 diagnostic group								
Leukemia	237	29.3	1	2	191	42.7	45	14.5
Lymphoma	165	20.4	2	3.9	117	26.2	46	14.8
CNS	110	13.6	4	7.8	7	1.6	99	31.9
Neuroblastoma	16	2.0	7	13.7	1	0.2	8	2.6
Retinoblastoma	8	1.0	2	3.9	2	0.5	4	1.3
Renal	40	5.0	8	15.7	14	3.1	18	5.8
Hepatic	4	0.5	0	0	3	0.7	1	0.3
Bone	53	6.6	1	2.0	35	7.8	17	5.5
Soft tissue	62	7.7	8	15.7	23	5.2	31	10.0
Germ cell	108	13.4	16	31.4	53	11.9	39	12.6
Other epithelial	5	0.6	2	3.9	1	0.2	2	0.7

Female survivors were twice as likely to have a hospital admission than males within NCSI level 1 where out of a total of 17 females, 15 (88.2%) had a recorded hospital admission, compared to 15 out of 34 males (44.1%). This gap became less pronounced with increasing NCSI levels (level 2: 124 out of 184 females (67.4%) had a recorded hospital admission compared to 144 of 263 males (54.8%)). Ninety out of 111 females (81.1%) within NCSI level 3 were admitted to hospital during the follow-up period, as were 147 out of 199 males (73.9%)). Of those that had at least 1 recorded hospital admission, there was little difference between females and males for time to first admission (years)—female 7.0 years (IQR 5.6–10.6), male 7.1 years (IQR 5.8–9.7), or for total length of hospital stay (days)—female 5.0 days (IQR 2.0–16.0). male 4.0 days (IQR 2.0–10.0).

### Comparison of hospital activity in those diagnosed in childhood (CH) versus young adults (YA)

There was no association between age category at diagnosis (0–17 years versus 18–29 years) and NCSI risk level (*p* = 0.70). The median number of admissions per person per year was similar for those diagnosed in childhood (0.23 IQR 0.13–0.50) compared with those diagnosed as young adults (0.36 IQR 0.15–0.82). When stratified by NCSI risk level, the median number of admissions per-person per year were again similar for those diagnosed in childhood compared with those diagnosed as young adults: NCSI level 2, CH 0.19 (IQR 0.11–0.34) YA 0.25 (IQR 0.13–0.48); NCSI level 3, CH 0.30 (IQR 0.17–0.68) YA 0.39 (IQR 0.21–0.86). NCSI level 1 was excluded from sub analysis as fewer than 5 individuals within this risk level (diagnosed as a YA) had a recorded hospital admission.

Of those who had at least one hospital admission, the median cumulative length of stay per person was similar for those diagnosed in childhood (CH: 5 days (IQR 2.0–12.0, *n* = 465 (68.7%))) compared with diagnosed as young adults (YA: 4 days (IQR 2.0–11.0, *n* = 70 (53.4%))). When stratified by NCSI risk levels, those diagnosed with cancer as YA follow a similar pattern of hospital usage compared with those diagnosed as a child. With increasing NCSI levels (levels 2 to 3), there was shorter time elapsed to first hospital admission and an increased cumulative length of stay per person (Table 2).

**Table 2:** Median and IQR for time to first admission and median length of stay of those 5-year cancer survivors whom had at least one hospital admission. Stratified by NCSI level, deprivation, and age at first cancer diagnosis (Childhood vs Young Adult)

	NCSI Risk Stratification Level							
	Level 1		Level 2	Level 3		All participants		
	*n*	Median (IQR)	*n*	Median (IQR)	*N*	Median (IQR)	*n*	Median (IQR)
Time to first hospital admission (years)	30	8.0 (6.0–12.2)	268	7.8 (5.8–11.0)	237	6.5 (5.5–8.4)	535	7.1 (5.7–9.8)
Age at cancer diagnosis								
Childhood (0–17 years)	28	8.4 (6.4–12.6)	241	8.0 (5.9–11.0)	196	6.5 (5.5–8.4)	465	7.1 (5.7–10.1)
Young adult (18–29 years)	2	-	27	.7 (5.9–11.0)	41	5.9 (5.5–7.7)	70	6.5 (5.6–8.4)
Townsend deprivation fifth								
1 and 2 (Least deprived)	7	6.5 (5.2–13.4)	38	8.1 (6.3–10.9)	45	6.3 (5.4–7.6)	90	6.7 (5.6–8.8)
3	4	-	69	8.2 (5.7–11.0)	54	6.6 (5.7–8.2)	127	7.3 (5.8–10.5)
4	7	11.6 (6.2–15.6)	53	7.2 (6.1–10.5)	51	5.8 (5.3–8.5)	111	6.7 (5.5–9.8)
5 (Most deprived)	12	8.2 (6.6–10.3)	108	8.0 (5.6–11.1)	87	6.9 (5.7–9.9)	207	7.4 (5.7–10.2)
Length of hospital stay (days) per person	30	3.5 (2.0–7.0)	268	4.0 (2–10.0)	237	6.0 (2.0–22.0)	535	5.0 (2.0–12.0)
Age at cancer diagnosis								
Childhood (0–17 years)	28	2.0 (2.0–4.0)	241	4.0 (2.0–10.0)	196	6.0 (3.0–23.0)	535	5.0 (2.0–12.0)
Young Adult (18–29 years)	2	-	27	2.0 (1.0–8.0)	41	7.0 (2.0–13.0)	70	4.0 (1.0–11.0)
Townsend deprivation fifth								
1 and 2 (Least deprived)	7	4.0 (2.0–7.0)	38	2.0 (1.0–7.0)	45	9.0 (5.0–28.0)	90	5.0 (2.0–18.8)
3	4	-	69	5.0 (2.0–12.0)	54	6.0 (2.0–19.0)	127	5.0 (2.0–12.0)
4	7	3.0 (2.0–7.0)	53	3.0 (1.0–13.0)	51	5.0 (2.0–15.0)	111	5.0 (2.0–13.0)
5 (Most deprived)	12	3.0 (2.0–8.5)	108	5.0 (2.0–10.5)	87	6.5 (3.0–19.0)	207	5.0 (2.0–12.0)

^1^*IQR* interquartile range, *NCSI* National Cancer Survivorship Initiative

^2^Some groups were excluded from analysis where *n* < 5

Poisson regression models demonstrate that for both YA and childhood cancers, those survivors in NCSI levels 1 (CH: IRR 0.32 (95% CI 0.26–0.41), YA: IRR 0.06 (95% CI 0.01–0.43)), and 2 (CH: IRR 0.46 (95% CI 0.42–0.50), YA: IRR 0.49 (95% CI 0.37–0.50)) are at significantly reduced risk of hospital admission compared with those in level 3, as well as significantly reduced length of stay, level 1; (CH: IRR 0.20 (95% CI 0.17–0.23), YA: IRR 0.05 (95% CI 0.12–0.20)), level 2; (CH: IRR 0.35 (95% CI 0.33–0.36), YA: IRR 0.49 (95% CI 0.40–0.58)) (Table 3).

**Table 3:** Results of Poisson regression models with IRRs (95% confidence interval) (controlling for sex and stratified by age at first cancer diagnosis (Childhood vs Young adult)) depicting the total number of hospital admissions and cumulative total length of hospital stay in a cohort of 5-year cancer survivors, presented by NCSI risk stratification level

	NCSI risk stratification level					
	Level 1		Level 2		Level 3	
	*n*	IRR (95% CI)	*n*	IRR (95% CI)	*n*	IRR (95% CI)
Total admissions						
All ages (*n* = 808)	51	0.31 (0.25–0.39)	447	0.46 (0.43–0.50)	310	1
Childhood (*n* = 694)	41	0.32 (0.26–0.41)	389	0.46 (0.42–0.50)	264	1
Young adult (*n* = 114)	10	0.06 (0.01–0.43)	58	0.49 (0.37–0.64)	46	1
Total length of stay						
All ages (*n* = 808)	51	0.20 (0.17–0.23)	447	0.36 (0.34–0.37)	310	1
Childhood (*n* = 694)	41	0.20 (0.17–0.23)	389	0.35 (0.33–0.36)	264	1
Young adult (*n* = 114)	10	0.05 (0.12–0.20)	58	0.49 (0.40–0.58)	46	1

^1^*IRR* incidence rate ratio, *NCSI* National Cancer Survivorship Initiative

#### Impact of deprivation on hospital activity

The proportion of survivors who had at least one hospital admission was similar across deprivation fifths (1st and 2nd = 62.1%, 3rd = 64.4%, 4th = 63.3%, and 5th = 65.6%). In those that had at least one admission, deprivation had little impact on the time to first admission or the median cumulative total length of stay, both across and within NCSI levels (Table 2).

There was a significant reduction in morbidity risk for the least deprived group within NCSI level 1 in terms of cumulative length of stay (IRR 0.55 (95% CI 0.38–0.81)) and for the least deprived group within NCSI level 2 in terms of number of admissions (IRR 0.60 (95% CI 0.48–0.74)) and cumulative length of stay (IRR 0.78 (95% CI 0.70–0.88)). Within NCSI level 3, there was a significant reduction in admission for deprivation fifths 3 and 4, IRR 0.80 (95% CI 0.70–0.92) and IRR 0.76 (95% CI 0.67–0.87) respectively.

There was no consistent evidence to support a significant association between deprivation level and the total number of admissions and length of stay, respectively (Table 4). Deprivation did not differentially impact the late-effect morbidity of male and female cancer survivors (*p* = 0.78), nor did it impact those diagnosed in childhood differently to those diagnosed as young adults (p = 0.45). When considering age at diagnosis as a continuous variable, the BIC when modelling with an interaction term between diagnosis age and deprivation was 6425, which was greater than when removing the interaction term, BIC = 6409.

**Table 4:** Results of sex- and-age adjusted Poisson regression models with IRRs (95% confidence intervals) to present the association between NCSI risk stratification levels and socioeconomic deprivation on the total number hospital admissions and total length of hospital admissions in a cohort of 5-year cancer survivors

	NCSI Risk Stratification Level							
	Level 1		Level 2		Level 3		All participants	
	*n*	IRR (95% CI)	*n*	IRR (95% CI)	*N*	IRR (95% CI)	*n*	IRR (95% CI)
Total admissions	51	0.31 (0.25–0.39)	447	0.46 (0.43–-0.50)	310	1		**-**
Townsend Deprivation fifth								
1 and 2 (Least deprived)	11	0.79 (0.44–1.12)	69	0.60 (0.48–0.74)	60	0.90 (0.79–1.02)	140	0.88 (0.79-0.98)
3	6	0.52 (0.22–1.23)	116	1.10 (0.95–1.27)	69	0.80 (0.70–0.92)	191	0.89 (0.81–0.97)
4	10	0.70 (0.38–1.31)	90	0.86 (0.73–1.02)	69	0.76 (0.67–0.87)	169	0.81 (0.73–0.90)
5 (Most deprived)	24	1	172	1	112	1	308	1
Total length of stay	51	0.20 (0.17–0.23)	447	0.36 (0.34–0.37)	112	1		**-**
Townsend deprivation fifth								
1 and 2 (Least Deprived)	11	0.55 (0.38-–.81)	69	0.78 (0.70–0.88)	60	1.10 (1.04–1.17)	140	1.15 (1.09–1.22)
3	6	0.65 (0.39–1.10)	116	1.42 (1.30–1.54)	69	0.81 (0.76–0.87)	191	0.95 (0.90–1.00)
4	10	0.69 (0.45–1.05)	90	1.00 (0.91–1.10)	69	0.69 (0.65–0.75)	169	0.80 (0.760.85)
5 (Most deprived)	24	1	172	1	112	1	308	1

^1^*IRR* incidence rate ratio, *NCSI* National Cancer Survivorship Initiative

^2^The most deprived quintile was the largest group across NCSI levels and was thus taken as the reference point for all models

#### Ethnicity

The median time to first admission was similar for those of White ethnic background—7.1 years (IQR 5.7–9.7*, n* = 483) and South Asians (SA)—7.2 (IQR 5.8–11.9*, n* = 44), as was the median number of admissions in those who had at least one: White—3.0 admissions (IQR 1.0–5.0, n = 483), SA—3.5 admissions (IQR 1.0–7.0, *n* = 44). The median cumulative length of stay per person was greater for SA—9.0 days (3.0–22.5) than White—5.0 days (2.0–12.0, *n* = 483), but not significantly so (Mann-Whitney test *p* = 0.94).

## Discussion

This study reports the first population-based evidence of the applicability of the NCSI risk-stratification model in predicting late-effect morbidity in cancer survivors diagnosed up to and including the age of 29 years, and to our knowledge, is the first to consider the impact of socio-economic deprivation and ethnicity within NCSI levels. Through utilization of hospital activity as a surrogate for morbidity burden, the study adds novel insights to the existing evidence base supporting the NCSI risk stratification model by demonstrating the effectiveness of its applicability to cancer survivors diagnosed in young adult life, aged 18–29 years inclusive.

Despite previous evidence supporting racial/ethnic disparities in mortality, with a disproportionate number of cancer deaths occurring among ethnic minorities, particularly African Americans [29] and previous work in long-term survivors of childhood cancer which found the morbidity associated with survival following cancer treatment to be greatest for the most socioeconomically deprived [30–32]; the study did not provide any strong consistent evidence to suggest that socioeconomic deprivation nor ethnicity independently impacted upon hospital activity, time to first admission, number of admissions, or length of hospital stay (Tables 2 and 4). Moreover, measures of deprivation did not contribute any significant variation in hospital activity within NCSI levels. This provides support for the three-tier NCSI model as a universally applicable tool for predicting late-effect morbidity in CYA cancer survivors, independent of socioeconomic position or ethnic group.

Females comprised 38.6% of the cohort, in line with previous work [33]. In keeping with previous work investigating hospital activity in long-term survivors [11] and general population studies [14], a higher proportion of female survivors were admitted to hospital. The sex-based disparities in admission rates were less pronounced for survivors within NCSI level 3. This is likely to be due to a consequential reduction in fertility in females who received higher intensity cancer treatment having fewer pregnancies and thus fewer pregnancy-related admissions to hospital [34].

The major strength of the current study lies in our capture of clinical outcome through exploitation of person-linked electronic inpatient hospital admission registry data. The hospital service provides insightful regional coverage of a geographic area which is not covered by any other pediatric oncology or specialist YA long-term follow-up service alike. Linkage of HES data to the cohort, which are drawn from a population-based register, removes the need to rely on subjective measures and thus reduces the scope for both self-report and selection bias.

However, using hospital admissions to estimate the burden of the adverse late effects of cancer treatment somewhat limits the extent to which we were able to quantify the effects of less severe issues faced by survivors which would only be captured through evaluation of primary care (General Practice) records. The number of patients within NCSI risk level 1 is lower than in previous studies investigating three-tier childhood cancer follow-up [9]. This results from the discharge of the lowest risk group, level 1, to primary care before they are 5 years from completion of therapy.

Further potential bias may have been introduced through failure of linkage of cancer survivors between the cancer registry and HES data and failure to capture private health care activity (thought to be under 2% of activity in the study age group [35]). Previous work linking the YSRCCYP to HES found that admission data was available for around 90% of the study population [36]. Reassuringly, this is comparable to other cancer registry linked HES admission studies [37] and compares favorably with studies based on questionnaire responses which typically have much lower response rates [38]. As such, whilst there may be some false and/or non-matches, the impact is likely to be small.

In conclusion, this study has generated novel evidence to support the efficacy of the three-tier NCSI risk stratification model’s applicability to young adults aged 18–29 years, in predicting their risk of late morbidity. Its adoption into clinical practice will support cancer services to provide evidence-based care with enhanced monitoring and support to those at risk and reassurance to those who are not as they live with and beyond cancer. Future population-based studies linking other registries, or national data, is needed to further validate the applicability of NCSI levels for use among different populations of cancer survivors, especially in those of non-South Asian ethnic backgrounds.

## Electronic supplementary material

ESM 1 (DOCX 34 kb)

## Data Availability

The data supporting the conclusions of this article are included within the article. The datasets generated and analyzed during the current study are not publicly available due to the potential for disclosure of individuals’ personal data.
