# Supplementary material for: Risk stratification of young adult survivors of cancer to estimate hospital morbidity burden: applicability of a pediatric therapy-based approach
Source: J Cancer Surviv. 2020 Sep 16;15(3):452–60. doi: 10.1007/s11764-020-00939-y (PMC8134299; doi:10.1007/s11764-020-00939-y)
Supplement: Supplementary file 1 — (DOCX 34 kb) [file 11764_2020_939_MOESM1_ESM.docx]

**Risk Stratification of Young Adult Survivors of Cancer to Estimate Hospital Morbidity Burden: Applicability of a Pediatric Therapy-Based Approach.**

Supplementary material

| **Type of Childhood Cancer** | | |  | | | | | | | |
| --- | --- | --- | --- | --- | --- | --- | --- | --- | --- | --- |
| **Treatment** | **HODG** | **NHL** | **CNS** | **NEURO** | **RETINO (heritable)** | **RETINO (non-heritable)** | **WILMS** | **BONE** | **STS** | **OTHER** |
| **S alone** | 1 | 1 | 2 | 1 | 2 | 1 | 1 | 1 | 1 | 1 |
| **R alone** | 3 | 2 | 3 | 2 | 3 | 2 | 2 | 2 | 2 | 2 |
| **C alone** | 2 | 2 | 2 | 2 | 2 | 1 | 2 | 2 | 2 | 2 |
| **S + R** | 3 | 2 | 3 | 2 | 3 | 2 | 2 | 2 | 2 | 2 |
| **S + C** | 2 | 2 | 2 | 2 | 2 | 1 | 2 | 2 | 2 | 2 |
| **R + C** | 3 | 2 | 3 | 3 | 3 | 2 | 3 | 3 | 3 | 2 |
| **S + R + C** | 3 | 2 | 3 | 3 | 3 | 2 | 3 | 3 | 3 | 2 |
| S- surgery; R - Radiotherapy; C - Chemotherapy; HODG - Hodgkin's lymphoma; NHL - Non-Hodgkin's lymphoma; CNS - Central Nervous System; NEURO - Neuroblastoma; RETINO - Retinoblastoma; STS - soft tissue sarcoma. | | | | | | | | | | |

**Figure S1.** NCSI Levels of clinical follow-up defined by type of childhood cancer and treatment received [1]. Abbreviations: S=surgery; R=Radiotherapy; C=Chemotherapy; HODG=Hodgkin's lymphoma; NHL=Non-Hodgkin's lymphoma; CNS=Central Nervous System; NEURO=Neuroblastoma; RETINO=Retinoblastoma; STS=soft tissue sarcoma.

**Table S1.** National Cancer Survivorship Initiative (NCSI) levels [2,3] stratified by Townsend Deprivation Index quintiles.

|  | NCSI Risk Stratification Level | | | | | |
| --- | --- | --- | --- | --- | --- | --- |
| 5-year Cancer Survivors | **Level 1** |  | **Level 2** |  | **Level 3** |  |
|  | **n** | **(%)** | **n** | **(%)** | **n** | **(%)** |
| Townsend Deprivation Quintile |  | |  | |  | |
| 1 (Least deprived) | 2 | 3.9 | 23 | 5.2 | 12 | 3.9 |
| 2 | 9 | 17.7 | 46 | 10.3 | 48 | 15.5 |
| 3 | 6 | 11.8 | 116 | 26.0 | 69 | 22.3 |
| 4 | 10 | 19.6 | 90 | 20.1 | 69 | 22.3 |
| 5 (Most deprived) | 24 | 47.1 | 172 | 38.5 | 112 | 36.1 |

**Table S2.** Townsend Deprivation Index quintiles (1-5) stratified by age group at initial cancer diagnosis (years).

|  | Townsend Deprivation Quintile | | | | | | | | | |
| --- | --- | --- | --- | --- | --- | --- | --- | --- | --- | --- |
| Age at Cancer Diagnosis (years) | **1 (Least deprived)** | | **2** |  | **3** |  | **4** |  | **5 (Most deprived)** | |
|  | **n** | **(%)** | **n** | **(%)** | **n** | **(%)** | **n** | **(%)** | **n** | **(%)** |
| 0-4 | 6 | 16.2 | 26 | 25.2 | 46 | 24.1 | 41 | 24.3 | 69 | 22.4 |
| 5-9 | 12 | 32.4 | 21 | 20.4 | 42 | 22.0 | 31 | 18.3 | 65 | 21.1 |
| 10-14 | 12 | 32.4 | 27 | 26.2 | 54 | 28.3 | 40 | 23.7 | 77 | 25.0 |
| 15-19 | 5 | 13.5 | 19 | 18.5 | 34 | 17.8 | 33 | 19.5 | 51 | 16.6 |
| 20-24 | 1 | 2.7 | 5 | 4.9 | 11 | 5.8 | 15 | 8.9 | 27 | 8.8 |
| 25-29 | 1 | 2.7 | 5 | 4.9 | 4 | 2.1 | 9 | 5.3 | 19 | 6.2 |

**Table S3.** National Cancer Survivorship Initiative (NCSI) levels [2,3] stratified by age group at initial cancer diagnosis (years). Pearson's chi-squared test*(χ^2^) P= 0.70.*

|  | NCSI Risk Stratification Level | | | | | |
| --- | --- | --- | --- | --- | --- | --- |
| Age at Cancer Diagnosis (years) | **Level 1** |  | **Level 2** |  | **Level 3** |  |
|  | **n** | **(%)** | **n** | **(%)** | **n** | **(%)** |
| 0-4 | 15 | 29.4 | 101 | 22.6 | 72 | 23.2 |
| 5-9 | 7 | 13.7 | 102 | 22.8 | 62 | 20.0 |
| 10-14 | 13 | 25.5 | 116 | 26.0 | 81 | 26.1 |
| 15-19 | 7 | 13.7 | 82 | 18.3 | 53 | 17.1 |
| 20-24 | 6 | 11.8 | 29 | 6.5 | 24 | 7.7 |
| 25-29 | 3 | 5.9 | 17 | 3.8 | 18 | 5.8 |
|  | | | | | | |

**References**

1. Frobisher C, Glaser A, Levitt GA, Cutter DJ, Winter DL, Lancashire ER, et al. Risk stratification of childhood cancer survivors necessary for evidence-based clinical long-term follow-up. BRITISH JOURNAL OF CANCER. 2017;117(11):1723-31.
2. NHS Improvement. Children and young people living with and beyond cancer. Designing and Implementing Pathways to Benefit Patient Aftercare: Continuing to Build the Evidence. 2011 [cited 2019 9/8/19]. Available from: [https://webarchive.nationalarchives.gov.uk/20130513172520/http://www.improvement.nhs.uk/LinkClick.aspx?fileticket=piHHerH%2fYd0%3d&tabid=56.](https://webarchive.nationalarchives.gov.uk/20130513172520/http:/www.improvement.nhs.uk/LinkClick.aspx?fileticket=piHHerH%2fYd0%3d&tabid=56.)
3. Glaser, A., Levitt, G., Morris, P., Tapp, J., & Gibson, F. (2013). Enhanced quality and productivity of long-term aftercare of cancer in young people. *Archives Of Disease In Childhood*, *98*(10), 818-824. doi: 10.1136/archdischild-2013-304348
